# C646 Protects Against DSS-Induced Colitis Model by Targeting NLRP3 Inflammasome

**DOI:** 10.3389/fphar.2021.707610

**Published:** 2021-07-12

**Authors:** Xueming Xu, Jing Li, Xiuyan Long, Sifan Tao, Xiaoyu Yu, Xixian Ruan, Kai Zhao, Li Tian

**Affiliations:** ^1^ Department of Hematology and Key Laboratory of Non-Resolving Inflammation and Cancer of Hunan Province, the Third Xiangya Hospital, Central South University, Changsha, China; ^2^ Department of Gastroenterology, the Third Xiangya Hospital, Central South University, Changsha, China

**Keywords:** C646, DSS-induced, NLRP3 inflammsome, NF-κB, ASC speck

## Abstract

Numerous pieces of evidence have identified that the NLRP3 inflammasome plays a pivotal role in the development and pathogenesis of colitis. Targeting the NLRP3 inflammasome represents a potential therapeutic treatment. Our previous studies have suggested that acetylation of NLRP3 is indispensable to NLRP3 inflammasome activation, and some acetyltransferase inhibitors could suppress the NLRP3 inflammasome activation. Here, we identified that C646, an inhibitor of histone acetyltransferase p300, exerts anti-inflammatory effects in DSS-induced colitis mice by targeting the NLRP3 inflammasome. Mechanistically, C646 not only inhibits NF-κB activation, leading to the decreased expression of pro-inflammatory cytokines (IL-1β, IL-6, and TNF-α) and NLRP3, but also suppresses the NLRP3 inflammasome assembly by disrupting the interaction between NLRP3 and ASC. In addition, C646 attenuated the LPS-induced acute systemic inflammation model. Thus, our results demonstrate the ability of C646 to suppress the NLRP3 inflammasome activity and its potential application in the treatment of inflammatory bowel disease.

## Introduction

Inflammatory bowel diseases (IBDs), comprising ulcerative colitis (UC) and Crohn’s disease (CD), are chronic and complex disorders of the gastrointestinal tract that are characterized by uncontrolled intestinal inflammation and epithelial injury. However, a comprehensive understanding of the pathophysiological mechanisms of IBD is far less clear (Kaser et al., 2010). Recent studies have suggested that biological therapies inhibition cytokines promote inflammation (e.g., anti–tumor necrosis factor TNF antibodies) or modulation of lymphocyte trafficking are effective in many IBD patients, implying that targeting dysregulated immune responses may be a potential therapeutic treatment in the future (Kaser et al., 2010; Kobayashi et al., 2020; Roda et al., 2020).

The NLRP3 inflammasome, a multiple protein complex, has been observed to play a critical role in the development and pathogenesis of IBD (Tourkochristou et al., 2019; Zhen and Zhang, 2019). It consists of the nucleotide-binding domain, leucine-rich repeat, pyrin domain–containing protein 3 (NLRP3), the apoptosis-associated speck-like protein containing a CARD (ASC) and caspase-1 (Martinon et al., 2009; Swanson et al., 2019). NLRP3 can be activated by a broad range of microbial motifs, endogenous danger signals, and environmental irritants. Upon activation, NLRP3 recruits ASC and caspase-1, leading to the maturation and secretion of IL-1β and IL-18, as well as gasdermin D–mediated pyroptotic cell death. It is well known that NLRP3 inflammasome activation is a two-step process, namely, priming and activation. Pathogen-associated molecule patterns (PAMPs), such as LPS, lead to the nuclear factor–κB (NF-κB) activation and increase the expression of NLRP3 and IL-1β, which is called the priming step. Following the priming step, diverse stimuli, such as ATP or nigericin, induce the assembly of the NLRP3 inflammasome and full activation. Recent studies have illustrated that excessive NLRP3 inflammasome–induced IL-1β aggravates colitis, and the absence of NLRP3 inflammasome components has a protective role in the dextran sodium sulfate (DSS)-induced colitis model (Tourkochristou et al., 2019; Zhen and Zhang, 2019). In addition, pharmacological inhibition of NLRP3 inflammasome activation ameliorates intestinal inflammation in animal models, suggesting that the NLRP3 inflammasome is a candidate target for treatment of IBD (Bauer et al., 2010; Du et al., 2017; Zhao Y. et al., 2019; Mei et al., 2019).

Our previous study (Zhao et al., 2019a; Liu et al., 2020) and the Danica Chen group’s research study (He et al., 2020) have both uncovered that acetylation of NLRP3 is required for the full activation of the NLRP3 inflammasome and have further suggested that some acetyltransferase inhibitors may suppress the NLRP3 inflammasome activation. By searching for kinds of lysine acetyltransferase inhibitors, we recently showed that SI-2 hydrochloride (SI-2) could block NLRP3 inflammasome activation (Liu et al., 2020). During the process, we noticed that one compound named C646, an inhibitor of histone acetyltransferase p300, not only decreased IL-1β but also TNF-α secretion. Whether C646 exerts a protective function in the colitis model is an interesting question to further study. In this work, we will evaluate the effect of C646 on colonic inflammation and explore the underlying mechanisms.

## Materials and Methods

### Reagents and Antibodies

Ultrapure lipopolysaccharide (LPS) (*E. coli* 0111:B4, cat. no. tlrl-3pelps), standard LPS (*E. coli* 0111:B4, cat. no. tlrl-eblps), ATP (cat. no. tlrl-atpl), nigericin (cat. no. tlrl-nig), and MSU (cat. no. tlrl-msu) were purchased from InvivoGen, lipofectamine 3,000 transfection reagent (cat. no. L3000015) was purchased from Thermo Fisher, C646 (S7152) was bought from Selleck Chemicals, mouse immunoglobin IgG protein (cat. no. ab198772) was purchased from Abcam, Protein A/G PLUS-Agarose (cat. no. sc-2003) was obtained from Santa Cruz, cell lysis buffer (CLB) (cat. no. 9803) was purchased from Cell Signaling Technology, and mouse IL-1β (cat. no. 88–7013), tumor necrosis factor-α (TNF-α) (cat. no. 88-7324), interleukin-6 (IL-6) (cat. no. 88-701364), and human IL-1β (cat. no. BMS22) ELISA kits were purchased from Thermo Fisher.

Anti–IL-1β (1:1,000, AF-401-NA; RRID:AB_416,684) was purchased from RD System, anti-NLRP3 (1:1,000, Cryo-2) and ASC (1:1,000, AL177) were purchased from Adipogen, anti–caspase-1 (1:1,000, ab179515), and anti-NEK7 (1:5,000, ab133514) were purchased from Abcam; Anti–β-actin (1:10,000, BH10D10), anti-p65 antibody (1:1,000, 8242), anti–p-p65 antibody (1:1,000, 3033), and GAPDH antibody (1:2,000, 5,174) were purchased from Cell Signaling Technology, and DyLight 488–labeled secondary antibody (1:50, A120-100D2) was purchased from InvivoGen.

### Mice

Wild-type (WT) C57BL/6 male mice (6–10 weeks old, 20–24 g) were obtained from Hunan SJA Laboratory Animal Co., Ltd. (Changsha, China), and they were bred under SPF conditions. Studies were conducted in accordance with the guidelines of the Institutional Animal Care and Use Committee of Central South University.

### Cell Culture

Primary peritoneal macrophages from C57BL/6 mice were harvested using the following method. Mice that were 6–8 weeks old were injected intraperitoneally with 3 ml of sterile 3% thioglycolate broth to elicit peritoneal macrophages. After 72 h, cells were collected *via* peritoneal lavage with 10–15 ml of RPMI medium 1,640 (Gibco). After being resuspended, the cells were cultured in RPMI-1640 containing 10% FBS, 100 U/ml penicillin, and 100 μg/ml streptomycin at 37ºC in a humidified incubator with 5% CO2.

### Inflammasome Activation

Mouse peritoneal macrophages were resuspended in 24-well (3–4 × $10^{5}$) or 6-well (2 × $10^{6}$) culture plates. To activate the inflammasome, macrophages were primed with LPS (100 ng/ml) for 3 h. Inflammasome stimulation was carried out as follows: 5 mM ATP, 10 μM nigericin for 1 h, and 200 μg/ml MSU for 6 h.

### Quantitative PCR

Total RNA was extracted by using the RNA Fast 200 kit according to the manufacturer’s instructions (FASTAGEN). Complementary DNA was reverse transcription synthesized by using TransScript All-in-One First-Strand cDNA Synthesis SuperMix for RtPCR (TransGen) according to the manufacturer’s protocols. Quantitative PCR was performed using SYBR Green (Vazyme Biotech) on a LightCycler 480 (Roche Diagnostics), and data were standardized to β-actin expression. The 2−ΔΔCT method was used to calculate relative expression changes. Gene-specific primers were as follows: NLRP3 forward, 5′-TGG ATG GGTTTG CTG GGA T-3′, reverse, 5′-CTG CGT GTA GCG ACT GTT GAG-3′; IL-1β forward, 5′- GCA ACT GTT CCT GAA CTC AAC T-3′ reverse, 5′-ATC TTT TGG GGT CCG TCA ACT-3′; IL-6 forward, 5′- TAG TCC TTCCTA CCC CAA TTT CC-3′ reverse, 5′- TTG GTC CTT AGC CAC TCC TTC-3′; TNF-α forward, 5′- GAC GTG GAA CTG GCA GAA GAG-3′ reverse, 5′-TTG GTG GTT TGT GAG TGT GAG-3′; β-actin forward, 5′-AGT GTGACG TTG ACA TCC GT-3′; and β-actin reverse, 5′-GCA GCT CAG TAA CAGTCC GC-3′.

### ASC Oligomerization

Primary peritoneal macrophage cells were treated with indicated stimuli. Next, the cells were lysed with Triton Buffer 50 mM Tris-HCl (pH 7.5), 150 mM NaCl, 0.5% Triton X-100, and 0.1 mM phenylmethylsulfonylfluoride (PMSF) for 10 min at 4°C. The cell lysates were centrifuged at 6000 g for 15 min at 4°C. After that, the supernatant was collected and resuspended in 200 μL Triton Buffer and 2 mM disuccinimidyl suberate (DSS). The complex was cross-linked for 30 min at 37°C. At last, cell pellets were washed twice. Samples were centrifuged, and the pellets were dissolved in sodium dodecyl sulphate (SDS) loading buffer for Western blotting.

### ASC Speck Formation

Primary peritoneal macrophage cells were seeded on chamber slides overnight. On the next day, the cells were treated with indicated stimuli. Next, the cells were washed three times with PBS buffer, fixed in 4% paraformaldehyde (PFA) for 10 min, permeabilized with 0.1% Triton X-100 for 15 min, and blocked with PBS buffer containing 3% BSA. Cells were then stained with anti-ASC (1:200) at 4°C for 12 h and with DyLight 488–labeled secondary antibody (1:50) at room temperature for 60 min. Last, DAPI was used to stain nuclei. Cells and tissues were visualized using a fluorescence microscope (Nikon Ti2-U).

### Immunoprecipitation and Western Blot

After stimulation, peritoneal macrophages were lysed in immunoprecipitation (IP) buffer containing 50 mM Tris HCl (pH 7.4), 50 mM EDTA, 200 mM NaCl, and 1% NP-40. Supplemented with a protease inhibitor cocktail, precleared cell lysates were then subjected to specific antibodies overnight and to protein G plus-agarose for 2 h and then washed four times with IP buffer. The immunoprecipitation complex was dissolved in sodium dodecyl sulphate (SDS) loading buffer for Western blotting.

For immunoblot analysis, cells were lysed with CLB supplemented with a protease inhibitor cocktail and PMSF, and then the cell lysates were centrifugated at 12,000 g for 5 min at 4°C. Equal amounts of extracts were separated by SDS-PAGE, and then they were transferred onto 0.22-mm PVDF membranes (Merck Millipore, ISEQ00010) for immunoblot analysis.

### SDD-AGE

The oligomerization of NLRP3 Western blot was analyzed following published protocols (Hou et al., 2011; Jiang et al., 2017). Cells were lysed with Triton X-100 lysis buffer and then centrifuged at 12,000 g for 5 min at 4°C. Next, the lysis was resuspended in 5 × sample buffer (2.5 × TBE, 50% glycerol, 10% SDS, and 0.0025% bromophenol blue) and run onto vertical 1.5% agarose gel. After electrophoresis for 1 h at a constant voltage of 80 V at 4°C in the running buffer (1 × TBE and 0.1% SDS), the proteins were transferred onto 0.22-mm PVDF membranes for immunoblotting.

### LPS-Induced Acute Systemic Inflammation

C57BL/6 mice were pretreated with or without C646 half an hour earlier. Next, they were injected intraperitoneally with LPS (20 mg/kg). After 8 h, the mice were terminated to collect the serum (Huang et al., 2020; Liu et al., 2020). The serum concentrations of IL-1β, TNF-α, and IL-6 were measured by ELISA, and the lungs were harvested.

### DSS-Induced Colitis

For acute experimental colitis induction, C57BL/6 mice were treated with 3% DSS or saline in their drinking water for 10 days. During the experiment, body weight, stool, and body posture were monitored daily to assess the DAI (Cooper et al., 1993; Alex et al., 2009). The DAI is the combined score of weight loss compared with initial weight, stool consistency, and bleeding. The details are as follows: (a) weight loss (0 point = none, 1 point = 1–5% weight loss, 2 points = 5–10% weight loss, 3 points = 10–15% weight loss, and 4 points = more than 15% weight loss), (b) stool consistency or diarrhea (0 point = normal, 2 points = loose stools, and 4 points = watery diarrhea), and (c) bleeding (0 point = no bleeding, 2 points = slight bleeding, and 4 points = gross bleeding). Mice were euthanized at the indicated time points, and the colon was immediately collected for colon length measurement, colon explant culture, colon Western blot analysis, and histological analysis.

### Colon Explant Culture

After removing the intestines from the mice, they were briefly washed with saline and then washed three times with cold RPMI-1,640 containing penicillin G (200 μg/ml) and streptomycin (200 μg/ml). The purpose is to wash away residual intestinal bacteria. Then they were incubated in supplemented culture medium containing penicillin G (200 μg/ml) and streptomycin (200 μg/ml). The medium was collected after incubation for 24 h at 37°C with 5% CO2, and the production of pro-inflammatory cytokines was determined by ELISA.

### Colonic Immunoblot Analysis

The mouse intestines and SDS lysate were lysed according to the ratio of 10 mg:200 ul, followed by ultrasound and grinding, after which they were centrifuged at 12,000 g for 10 min, and the supernatant was aspirated. After protein quantitative analysis, extracts were boiled and dissolved in sodium dodecyl sulphate (SDS) loading buffer for Western blotting.

### Histological Analysis

The mouse tissue was fixed in 4% PFA at 4°C for 24 h and sectioned after being embedded in paraffin. The sections were prepared and stained with H&E using standard procedures. The slides were inspected under a Nikon ECL IPSE Ci biological microscope, and images were captured using a Nikon DS-U3 color digital camera.

### Statistical Analysis

All the results are expressed as the mean ± SD, and statistical analyses were performed using GraphPad Prism software 8.0. Statistical analysis was performed using the ANOVA with the Bonferroni test for the two groups test. *p* > 0.05 was not a significant statistical difference; *p* < 0.05 was considered statistically significant, with increasing levels of confidence displayed as **p < 0.05; ***p < 0.01; ****p < 0.0001.

## Results

### C646 Protects Against DSS-Induced Colitis Murine Model

We previously (Liu et al., 2020) showed that C646 may suppress inflammation *in vitro*. To further study the effect of C646 on the treatment of colonic inflammation, the DSS-induced acute colitis murine model was adopted, which could destruct epithelial cells, increase the permeability of the colon, and trigger inflammation in the gut (Alex et al., 2009).

**FIGURE 1:**
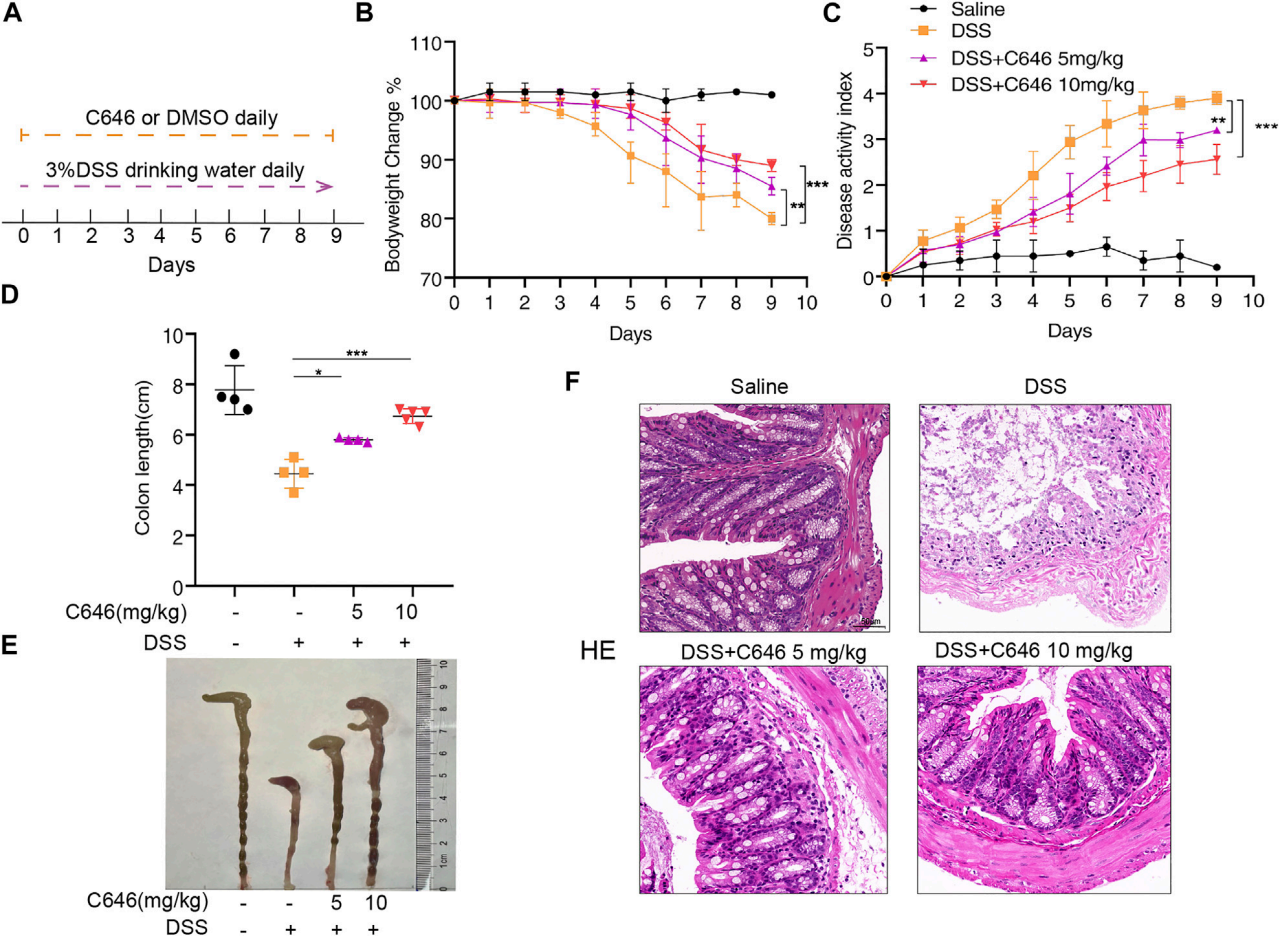
C646 ameliorates symptoms in the DSS-induced colitis mouse model. C57BL/6 mice were administrated with 3% DSS or water for 10 days. C646 (5 or 10 mg/kg) was given by intraperitoneal injection from day 0 to day 9. **(A)** Experimental design of the DSS-induced colitis mice model and C646 treatment. **(B)** Changes in the mice’s body weight were measured and presented as a percent of body weight change (*n* ≥ 4 per group). **(C)** DAI was calculated (*n* ≥ 4 per group). **(D)** and **(E)** Gross morphology of colons from C57BL/6 mice. The lengths of the colons were measured on day 9 (*n* ≥ 4). **(F)** H&E-stained images of colon sections fed with DSS and then treated with or without C646. Scale bar, 50 μm.

Mice received 3% DSS in their drinking water for 10 days, and C646 was given once a day by intraperitoneal injection from day 0 to day 9 (Figure 1A). The clinical assessments, including body weight, grossly bloody stools, and stool consistency, were recorded daily. As shown in Figure 1B, mice only administrated with DSS suffered a 20% weight loss, while for the group treated with 5 mg/kg or 10 mg/kg of C646, it improved the weight loss caused by DSS significantly. The disease activity index (DAI) is the summary of scores including weight loss, diarrhea, and gross bleeding, used to assess the severity of colitis. Similarly, C646 reduced the DAI in a dose-dependent manner (Figure 1C). Colon length is another indicator for monitoring the severity of colitis, and we found that C646-treated mice showed longer colon length than the control group (Figures 1D,E). To further evaluate the effect of C646 in colitis, histological examination of hematoxylin and eosin (H&E) staining was carried out. We observed severe mucosal damage, gland destruction, and infiltration of mononuclear cells in the colon specimens of DSS-fed mice. In contrast, C646-treated mice exhibited intact colonic architecture without mucosal damage and with less mononuclear cell infiltration (Figure 1F). Thus, our results demonstrated that C646 protects against DSS-induced colitis.

### C646 Suppresses NLRP3 Inflammasome Activity in DSS-Induced Colitis Model

**FIGURE 2:**
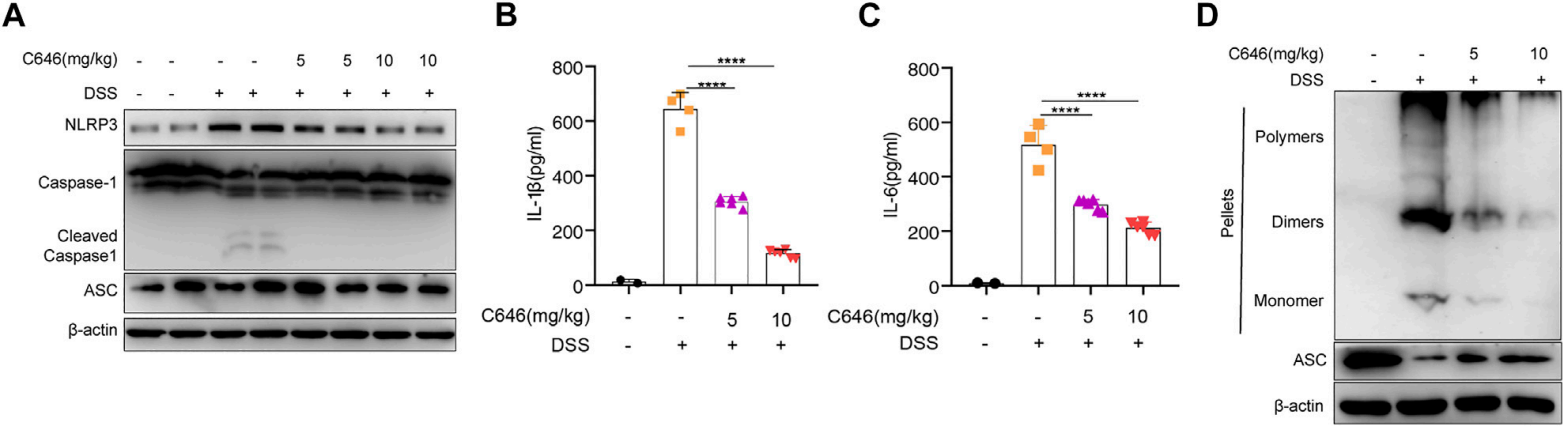
C646 suppresses NLRP3 inflammasome activity in the DSS-induced colitis model. **(A)** Immunoblot analysis of cleaved caspase 1 and NLRP3. Data were normalized to the expression of β-actin as reference. **(B)** and **(C)** Protein level of the cleaved IL-1β and IL-6 in colon homogenates was determined by ELISA. **(D)** Immunoblot analysis of ASC oligomerization in cross-linked cytosolic pellets of DSS-induced colitis treated with C646 or DMSO. The results of **(B)** and **(C)** were expressed as mean ± SD. Statistics were analyzed using one-way ANOVA and the Bonferroni test. **p < 0.05; ***p < 0.01; ****p < 0.0001.

Numerous studies (Bauer et al., 2010; Tourkochristou et al., 2019; Zhen and Zhang, 2019) have suggested that the NLRP3 inflammasome is a potential target for the treatment of colitis. Then we examined whether C646 could inhibit NLRP3 inflammasome activity in the DSS-induced model. We detected the expression of NLRP3 inflammasome components and the secretion of IL-1β in colon tissue. We observed that C646 markedly reduced the expression of NLRP3 and the cleavage of caspase-1, the downstream event of inflammasome activation (Figure 2A). Consistently, IL-1β secretion from the C646-treated group was decreased in a dose-dependent manner (Figure 2B), and IL-6 also showed a similar pattern (Figure 2C). ASC speck formation was another hallmark of NLRP3 inflammasome activation, and we further detected the ASC speck by using disuccinimidyl suberate cross-linking (Fernandes-Alnemri et al., 2007; Davis et al., 2011). As anticipated, C646 reduced ASC speck formation in colon tissues (Figure 2D). Taken together, these data suggested that C646 inhibits NLRP3 inflammasome activation in the DSS-induced colitis model.

### C646 Inhibits NLRP3 Inflammasome at Both Priming and Assembly Steps

**FIGURE 3:**
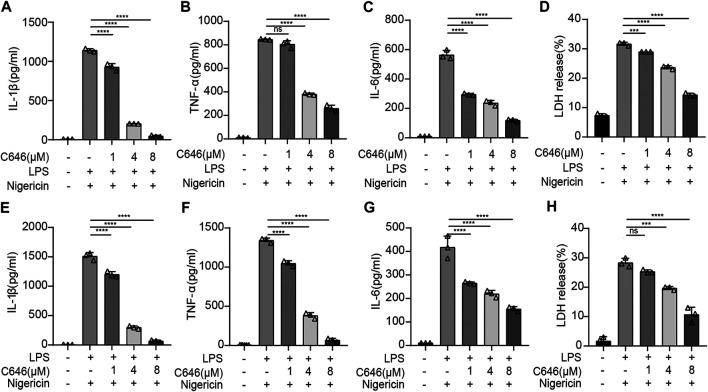
C646 suppresses the NLRP3 inflammasome at the priming and assembly steps.**(A–D)** Mouse peritoneal macrophages were treated with C646 and then primed with LPS, followed by stimulation with nigericin. The supernatant (SN) was subjected to ELISA assay for IL-1β, IL-6, TNF-α, and LDH. **(E–H)** Mouse peritoneal macrophages were primed with LPS and then treated with C646, followed by stimulation with nigericin. The supernatant (SN) was subjected to ELISA assay for IL-1β, IL-6, TNF-α, and LDH. For **(A)** to **(H),** values are mean ± SD. Statistics were analyzed using one-way ANOVA and the Bonferroni test. **p < 0.05; ***p < 0.01; ****p < 0.0001.

To further study the inhibiting effect of C646 on the NLRP3 inflammasome, we treated primary macrophages with C646 before or after LPS priming and then stimulated them with nigericin, an NLRP3 inflammasome agonist. We observed that the secretion of IL-1β, TNF-α, and IL-6 was decreased whenever treatment with C646 was carried out and so was cell death, evaluated by LDH release (Figures 3A–H), suggesting that C646 suppresses NLRP3 inflammasome activation at both priming and activation steps. We also noticed that C646 inhibited nigericin-induced NLRP3 activation in a dose-dependent manner and achieved the maximum suppressing effect at 8 μM.

**FIGURE 4:**
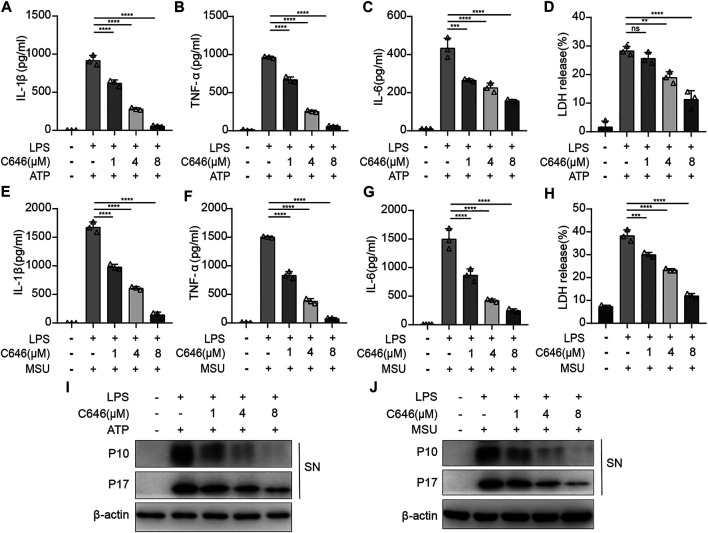
C646 suppresses the NLRP3 inflammasome activated by different agonists. **(A–H)** ELISA assay for IL-1β, IL-6, TNF-α, and LDH in the supernatant of mouse peritoneal macrophages that were treated with C646 or DMSO upon stimulation of LPS + ATP or LPS + MSU. **(I)** and **(J)** Mouse peritoneal macrophages were primed with LPS and then treated with C646, followed by stimulation with ATP or MSU. The supernatant (SN) was subjected to Western blot analysis for the indicated protein levels. Data are representative of three independent experiments. For **(A)** to **(H),** values are mean ± SD. Statistics were analyzed using one-way ANOVA and the Bonferroni test. **p < 0.05; ***p < 0.01; ****p < 0.0001.

To further confirm the results, ATP and MSU, another two kinds of NLRP3 inflammasome agonists, were used. C646 had a similar suppressing effect on ATP- or MSU-induced NLRP3 inflammasome activation, reflected by cytokine production and cell death (Figures 4A–H). In addition, the cleavage of caspase-1 and pro–IL-1β detected by the Western blot was shown to be decreased upon C646 treatment (Figures 4I,J). Thus, these data indicated that C646 inhibits NLRP3 inflammasome activation at both priming and activation steps.

### C646 Inhibits the Activation of the NF-κB Pathway

**FIGURE 5:**
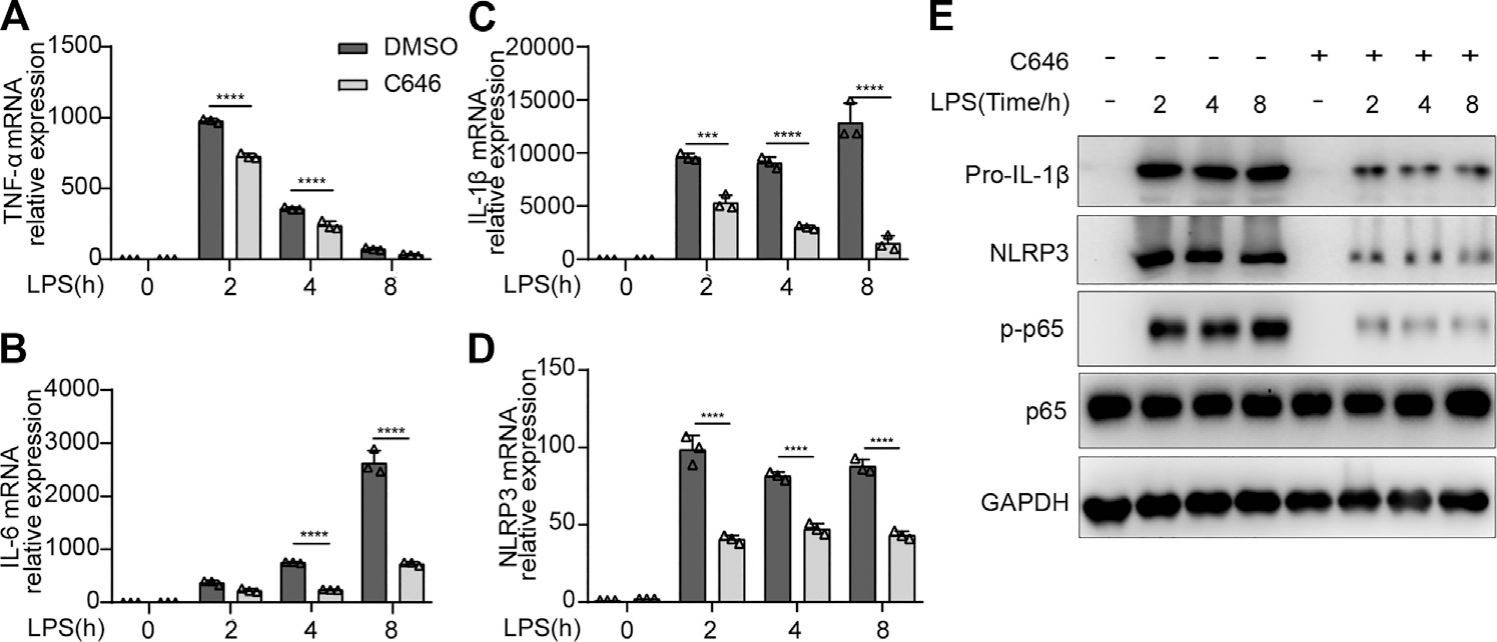
C646 inhibits the activation of the NF-κB signaling. **(A–D)** Quantitative PCR analysis of mRNA expression of TNF-α, IL-6, IL-1β, and NLRP3 in primary peritoneal macrophages treated with C646 and primed with LPS for the indicated hours. **(E)** Immunoblot analysis of p-p65, p65, NLRP3, and IL-1β in mouse peritoneal macrophages treated with C646 and then primed with LPS for the indicated hours. Values are mean ± SD. For **(A)** to **(D),** values were analyzed using two-way ANOVA and the Bonferroni test. *p < 0.05, **p < 0.01 and ***p < 0.001.

Next, we decided to explore whether the mRNA expressions of pro-inflammatory cytokines were impaired by C646 treatment. As expected, C646 significantly inhibited the mRNA levels of IL-1β, TNF-α, and IL-6, as well as NLRP3 (Figures 5A–D). Since NF-κB signaling dominantly contributed to the production of pro-inflammatory cytokines and NLRP3 (Bauernfeind et al., 2009), we detected this pathway and observed that C646 sharply decreased the phosphorylation of p65, a key event for NF-κB activation (Figure 5E). Consistently, the protein expression of NLRP3 and pro–IL-1β was also decreased upon C646 treatment (Figure 5E). Thus, these data suggested that C646 inhibits the NF-κB pathway activation, which is consistent with a previous study (Fang et al., 2019).

### C646 Inhibits NLRP3 Inflammasome Activation by Disrupting the Association Between NLRP3 and ASC

**FIGURE 6:**
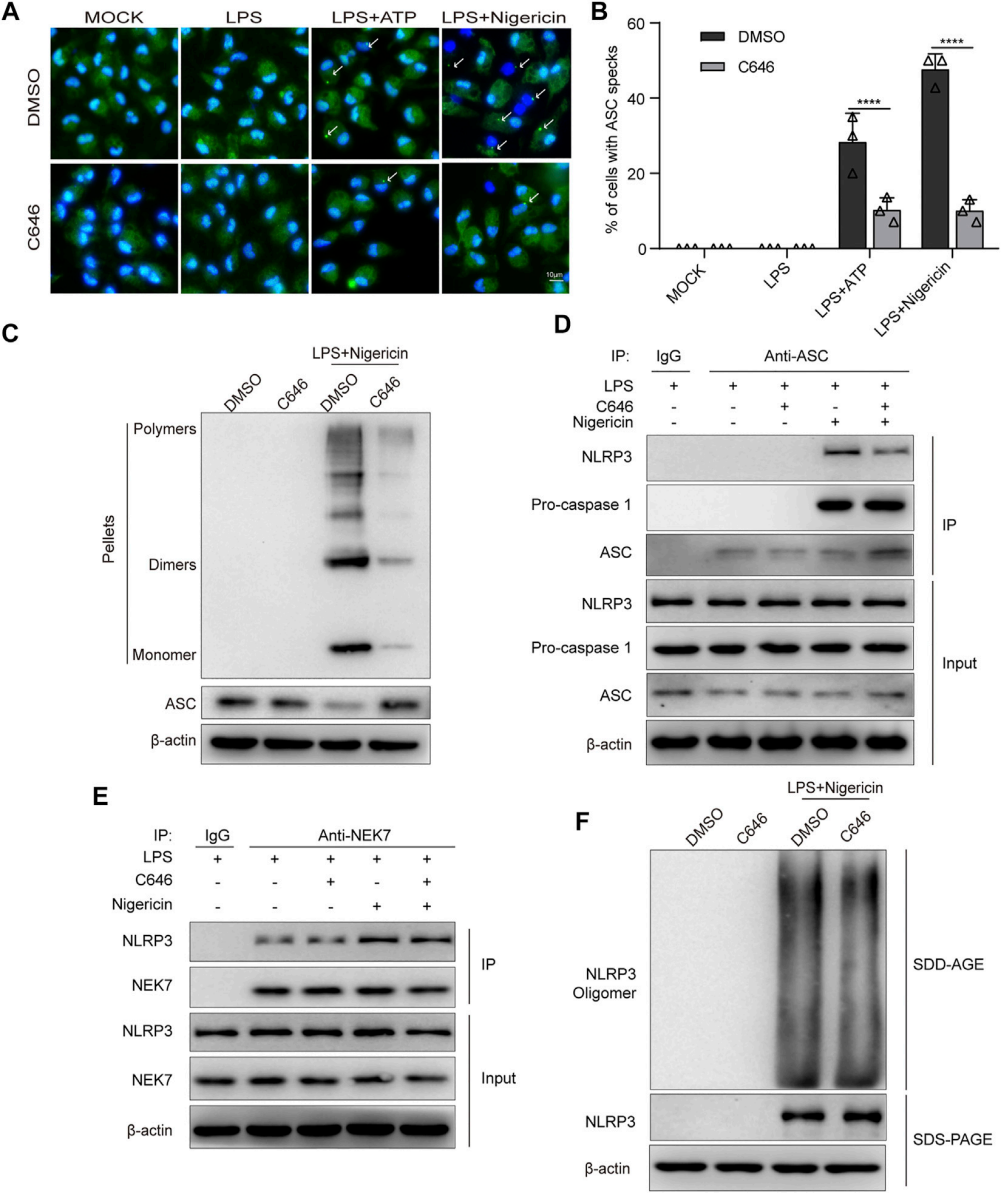
C646 inhibits NLRP3 inflammasome assembly. **(A–B)** Immunofluorescence microscopy analysis of primary macrophages treated with LPS–ATP or LPS–nigericin with or without C646 treatment. Representative images of ASC speck subcellular distribution **(A)** and quantification of ASC speck formation **(B)** by the number of cells with ASC specks. Scale bar, 10 μm. **(C)** Immunoblot analysis of ASC oligomerization in cross-linked cytosolic pellets of LPS-primed primary macrophages treated with C646 or DMSO and then stimulated with nigericin. **(D)** Immunoblot analysis of the interaction of NLRP3 and ASC in LPS-primed primary macrophages treated with C646 and then stimulated with nigericin. **(E)** Immunoblot analysis of the interaction of NEK7 and NLRP3 in LPS-primed primary macrophages treated with C646 and then stimulated with nigericin. **(F)** Immunoblot analysis of NLRP3 oligomerization using SDD-AGE or SDS-PAGE assays in LPS-primed primary macrophages treated with C646 and then stimulated with nigericin. **(D)** and **(E)** Data shown are representative of three independent experiments. Values are mean ± SD. Statistics of **(B)** were analyzed using two-way ANOVA and the Bonferroni test. **p < 0.05; ***p < 0.01; ****p < 0.0001.

To explore the underlying mechanism of C646 inhibiting the NLRP3 inflammasome at the activation step, we first checked ASC speck formation. We found that C646 treatment significantly attenuated the percentage of cells containing ASC specks in the ATP or nigericin stimulation groups *via* immunofluorescence (Figures 6A,B), and this was further demonstrated by the detection of ASC oligomerization using disuccinimidyl suberate cross-linking (Figure 6C). Thus, C646 inhibits the formation of ASC specks.

Previous studies have suggested that interaction between NLRP3 and ASC is an upstream step for ASC speck formation. By using immunoprecipitation and immunoblotting assays, we found that C646 significantly inhibits the interaction between NLRP3 and ASC but not ASC and caspase-1 upon LPS + nigericin treatment (Figure 6D). Recent work has suggested that NEK7, a member of the mammalian NIMA-related kinases, is essential for the activation of the NLRP3 inflammasome by interacting with NLRP3 and promoting ASC oligomerization (Franklin et al., 2014; He et al., 2016; Schmid-Burgk et al., 2016). Therefore, we investigated the NLRP3–NEK7 interaction and observed that C646 barely affected the association between them (Figure 6E). Since oligomerized NLRP3 can recruit ASC (Swanson et al., 2019), we further explored whether C646 affects NLRP3 oligomerization, as the results showed that C646 had no effect on the oligomerization of NLRP3 (Figure 6F). Overall, our results indicated that C646 disrupts the interaction between NLRP3 and ASC and then leads to the impairment of NLRP3 inflammasome assembly.

### C646 Ameliorates LPS-Induced Acute Systemic Inflammation *in vivo*

**FIGURE 7:**
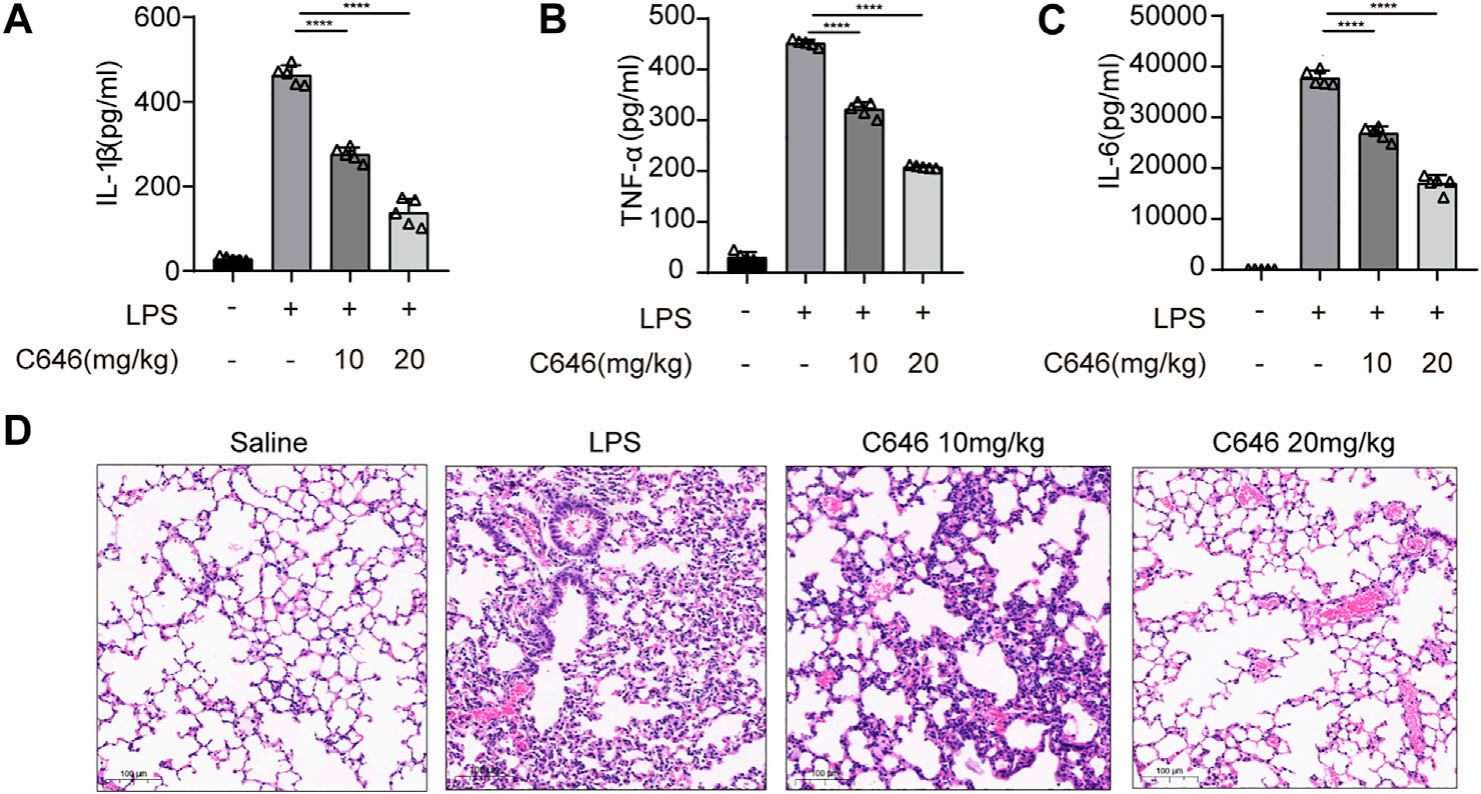
C646 ameliorates LPS-induced acute systemic inflammation *in vivo.* C57BL/6 mice were given intraperitoneal (i.p.) injection of N.S or C646 (10 or 20 mg/kg) 30 min before i. p. injection of LPS (20 mg/kg) for 8 h (n = 3–5/group). ELISA of serum IL-1β **(A)**, IL-6 **(B)**, and TNF-α **(C)**. Representative H&E images of lung sections **(D)** were collected. Scale bar, 100 μm. For **(A)** to **(C),** values were analyzed using one-way ANOVA and the Bonferroni test. **p < 0.05; ***p < 0.01; ****p < 0.0001.

We further verified the function of C646 in another inflammation model by adopting LPS-induced acute systemic inflammation (Mariathasan et al., 2006). Mice were pretreated with C646 (10 or 20 mg/kg i. p.) or saline and then challenged with LPS (20 mg/kg). As shown, the C646-treatment group decreased IL-1β, IL-6, and TNF-α levels in serum in a dose-dependent manner (Figures 7A–C). Furthermore, C646 alleviated the endotoxemia-induced lung injury, as evaluated by histopathology (Figure 7D). Thus, C646 shows therapeutic effects on LPS-induced acute systemic inflammation.

## Discussion

In the present study, we have found that C646 plays an anti-inflammatory role in the DSS-induced colitis model by targeting the NLRP3 inflammasome. Mechanistically, C646 not only impairs NLRP3 and pro-inflammatory cytokine expression by affecting NF-κB activation at the priming step but also decreases NLRP3–ASC association, and then it inhibits the NLRP3 inflammasome assembly at the activation step. In addition, C646 ameliorates LPS-induced acute systemic inflammation. Thus, our study demonstrated that C646 could be a potential agent for the treatment of colitis.

C646, an inhibitor of histone acetyltransferase p300, was initially discovered by virtual ligand screening (Bowers et al., 2010). After that, the potential application of C646 was mostly evaluated in cancer biology (Oike et al., 2014; Wu et al., 2015; Ono et al., 2016; Liang et al., 2020). Numerous studies have found that C646 could induce apoptosis, arrest the cell cycle, and block the proliferation in cancer cells (Oike et al., 2014; Wu et al., 2015). Recent works have suggested an immunomodulatory role of C646 in antibacterial (Fang et al., 2019) and antiviral (Zhao et al., 2015) responses. Moreover, the application of C646 has been enhanced in inflammatory lung diseases. Based on our previous study (Zhao K. et al., 2019; Liu et al., 2020), we investigated the anti-inflammatory role of C646 in the DSS-induced colitis model. We found that C646 improved the weight loss, diarrhea, and gross bleeding induced by DSS. Moreover, the C646-treated group shows longer colon length and exhibits intact colonic architecture without mucosal damage and with less mononuclear cell infiltration in comparison to the control group, suggesting a protective role of C646 in the DSS-induced murine model. Then we explored whether C646 affects the NLRP3 inflammasome activation in colon tissues. We observed that DSS induced elevated expression of NLRP3 and cleavage of caspase-1, which were attenuated by C646 treatment in colon tissues. The following secretion of IL-1β and IL-6 was also decreased. Consistently, ASC speck formation, an NLRP3 inflammasome activation marker, was impaired upon C646 treatment, indicating that C646 inhibits NLRP3 inflammasome activation in the DSS-induced murine model. We further investigated the underlying mechanism of C646 on the NLRP3 inflammasome in primary macrophages. We found that C646 inhibits NLRP3 inflammasome activity in a dose-dependent manner, and this suppressing effect was achieved both at the priming step and the activation step when given C646 before or after LPS priming. Consistent with early studies (Atreya et al., 2008), our study also observed that C646 inhibits NF-κB–induced gene expression in primary macrophages, which could be explained by the role of p300 in the regulation of the NF-κB pathway. Moreover, we found that C646 inhibits NLRP3 inflammasome assembly by disrupting the association between NLRP3 and ASC but not NLRP3 and NEK7. Since oligomerized NLRP3 is required for recruiting ASC, we further investigated this process and observed that C646 has no effect on the oligomerization of NLRP3. We speculated that C646 may interact with NLRP3 or ASC or both of them and then block the association between them. This hypothesis may need the help of structural biology to be demonstrated, which still needs further investigation. In addition, we confirmed the anti-inflammatory function of C646 in another LPS-induced systemic inflammation model *in vivo*. Thus, C646 shows a robust suppressing effect on the NLRP3 inflammasome.

In colitis animal models, the NLRP3 inflammasome has been regarded to have both pathogenic and protective effects (Tourkochristou et al., 2019; Zhen and Zhang, 2019). The studies by Bauer et al. (Bauer et al., 2010; Bauer et al., 2012; Mak'Anyengo et al., 2018) showed that mice lacking NLRP3 exhibited attenuated colitis compared to control mice in both DSS- and TNBS (2,4,6-trinitrobenzene sulfonic acid)-induced models. In contrast, other studies (Castro-Dopico et al., 2019) have found that mice with NLRP3, ASC, or caspase-1 deficiency exhibited more severe experimental colitis and decreased intestinal epithelial integrity, suggesting a protective role of the NLRP3 inflammasome. Although the contradictory results in mice models exist, human data have shown that NLRP3 inflammasome activity is increased in UC and CD patients, and some studies even demonstrated that targeting NLRP3 activity, such as MCC950 (Bauer et al., 2010), flavonoid VI-16 (Zhao et al., 2019), carboxyamidotriazole (CAI) (Du et al., 2017), and PAP-1 (Mei et al., 2019), in IBD murine models displayed therapeutic effects. In this study, we have found that C646 suppresses NLRP3 inflammasome activity through impairing the NF-κB pathway at the priming step and inhibiting the assembly at the activation step. It is well known that the NF-κB pathway is crucial for the production of inflammatory cytokines, including IL-6 and TNF-α, both of which are important targets for the treatment of intestinal inflammation (Mao et al., 2018; Waljee et al., 2018). Considering that the use of curcumin (Gong et al., 2015; Iqbal et al., 2018; Wang et al., 2018), an agent inhibiting NLRP3 inflammasome and NF-κB activation, in UC patients was linked to clinical improvement and endoscopic remission, C646 may have potential in clinical studies for IBD treatment, but we still need more clinical data to test.

Overall, our study showed that C646 could ameliorate DSS-induced colitis, expanding the anti-inflammatory potential of this agent. Moreover, the suppressing effect on the NLRP3 inflammasome delineates a new insight into the immunomodulatory function of C646.

## Conclusion

In summary, our study indicates the protective effect of C646 in DSS-induced colitis. This effect could be mediated by suppressing the activation of the NLRP3 inflammasome and the NF-κB signaling pathways. Our results demonstrated that C646 could be a candidate agent for IBD treatment.

## Data Availability

The original contributions presented in the study are included in the article/Supplementary Material; further inquiries can be directed to the corresponding authors.

## References

[B1] Alex P. Zachos N. C. Nguyen T. Gonzales L. Chen T.-E. Conklin L. S. (2009). Distinct Cytokine Patterns Identified from Multiplex Profiles of Murine DSS and TNBS-Induced Colitis. Inflamm. Bowel Dis. 15, 341–352. 10.1002/ibd.20753 18942757 PMC2643312

[B2] Atreya I. Atreya R. Neurath M. F. (2008). NF-κB in Inflammatory Bowel Disease. J. Intern. Med. 263, 591–596. 10.1111/j.1365-2796.2008.01953.x 18479258

[B3] Bauer C. Duewell P. Lehr H.-A. Endres S. Schnurr M. (2012). Protective and Aggravating Effects of Nlrp3 Inflammasome Activation in IBD Models: Influence of Genetic and Environmental Factors. Dig. Dis. 30 (Suppl. 1), 82–90. 10.1159/000341681 23075874

[B4] Bauer C. Duewell P. Mayer C. Lehr H. A. Fitzgerald K. A. Dauer M. (2010). Colitis Induced in Mice with Dextran Sulfate Sodium (DSS) Is Mediated by the NLRP3 Inflammasome. Gut 59, 1192–1199. 10.1136/gut.2009.197822 20442201

[B5] Bauernfeind F. G. Horvath G. Stutz A. Alnemri E. S. Macdonald K. Speert D. (2009). Cutting Edge: NF-κB Activating Pattern Recognition and Cytokine Receptors License NLRP3 Inflammasome Activation by Regulating NLRP3 Expression. J. Immunol. 183, 787–791. 10.4049/jimmunol.0901363 19570822 PMC2824855

[B6] Bowers E. M. Yan G. Mukherjee C. Orry A. Wang L. Holbert M. A. (2010). Virtual Ligand Screening of the P300/CBP Histone Acetyltransferase: Identification of a Selective Small Molecule Inhibitor. Chem. Biol. 17, 471–482. 10.1016/j.chembiol.2010.03.006 20534345 PMC2884008

[B7] Castro-Dopico T. Dennison T. W. Ferdinand J. R. Mathews R. J. Fleming A. Clift D. (2019). Anti-commensal IgG Drives Intestinal Inflammation and Type 17 Immunity in Ulcerative Colitis. Immunity 50, 1099–1114 e10. 10.1016/j.immuni.2019.02.006 30876876 PMC6477154

[B8] Cooper H. S. Murthy S. N. Shah R. S. Sedergran D. J. (1993). Clinicopathologic Study of Dextran Sulfate Sodium Experimental Murine Colitis. Lab. Invest. 69, 238–249. 8350599

[B9] Davis B. K. Wen H. Ting J. P.-Y. (2011). The Inflammasome NLRs in Immunity, Inflammation, and Associated Diseases. Annu. Rev. Immunol. 29, 707–735. 10.1146/annurev-immunol-031210-101405 21219188 PMC4067317

[B10] Du X. Chen W. Wang Y. Chen C. Guo L. Ju R. (2017). Therapeutic Efficacy of Carboxyamidotriazole on 2,4,6-trinitrobenzene Sulfonic Acid-Induced Colitis Model Is Associated with the Inhibition of NLRP3 Inflammasome and NF-κB Activation. Int. Immunopharmacol. 45, 16–25. 10.1016/j.intimp.2017.01.015 28152446

[B11] Fang F. Li G. Jing M. Xu L. Li Z. Li M. (2019). C646 Modulates Inflammatory Response and Antibacterial Activity of Macrophage. Int. Immunopharmacol. 74, 105736. 10.1016/j.intimp.2019.105736 31302452

[B12] Fernandes-Alnemri T. Wu J. Yu J.-W. Datta P. Miller B. Jankowski W. (2007). The Pyroptosome: a Supramolecular Assembly of ASC Dimers Mediating Inflammatory Cell Death via Caspase-1 Activation. Cell Death Differ 14, 1590–1604. 10.1038/sj.cdd.4402194 17599095 PMC3345951

[B13] Franklin B. S. Bossaller L. De Nardo D. Ratter J. M. Stutz A. Engels G. (2014). The Adaptor ASC Has Extracellular and 'prionoid' Activities that Propagate Inflammation. Nat. Immunol. 15, 727–737. 10.1038/ni.2913 24952505 PMC4116676

[B14] Gong Z. Zhou J. Li H. Gao Y. Xu C. Zhao S. (2015). Curcumin Suppresses NLRP3 Inflammasome Activation and Protects against LPS-Induced Septic Shock. Mol. Nutr. Food Res. 59, 2132–2142. 10.1002/mnfr.201500316 26250869

[B15] He M. Chiang H.-H. Luo H. Zheng Z. Qiao Q. Wang L. (2020). An Acetylation Switch of the NLRP3 Inflammasome Regulates Aging-Associated Chronic Inflammation and Insulin Resistance. Cel Metab. 31, 580–591. e585. 10.1016/j.cmet.2020.01.009 PMC7104778 32032542

[B16] He Y. Zeng M. Y. Yang D. Motro B. Núñez G. (2016). NEK7 Is an Essential Mediator of NLRP3 Activation Downstream of Potassium Efflux. Nature 530, 354–357. 10.1038/nature16959 26814970 PMC4810788

[B17] Hou F. Sun L. Zheng H. Skaug B. Jiang Q.-X. Chen Z. J. (2011). MAVS Forms Functional Prion-like Aggregates to Activate and Propagate Antiviral Innate Immune Response. Cell 146, 448–461. 10.1016/j.cell.2011.06.041 21782231 PMC3179916

[B18] Huang L. Luo R. Li J. Wang D. Zhang Y. Liu L. (2020). β-Catenin Promotes NLRP3 Inflammasome Activation via Increasing the Association between NLRP3 and ASC. Mol. Immunol. 121, 186–194. 10.1016/j.molimm.2020.02.017 32244067

[B19] Iqbal U. Anwar H. Quadri A. A. (2018). Use of Curcumin in Achieving Clinical and Endoscopic Remission in Ulcerative Colitis: A Systematic Review and Meta-Analysis. Am. J. Med. Sci. 356, 350–356. 10.1016/j.amjms.2018.06.023 30360803

[B20] Jiang H. He H. Chen Y. Huang W. Cheng J. Ye J. (2017). Identification of a Selective and Direct NLRP3 Inhibitor to Treat Inflammatory Disorders. J. Exp. Med. 214, 3219–3238. 10.1084/jem.20171419 29021150 PMC5679172

[B21] Kaser A. Zeissig S. Blumberg R. S. (2010). Inflammatory Bowel Disease. Annu. Rev. Immunol. 28, 573–621. 10.1146/annurev-immunol-030409-101225 20192811 PMC4620040

[B22] Kobayashi T. Siegmund B. Le Berre C. Wei S. C. Ferrante M. Shen B. (2020). Ulcerative Colitis. Nat. Rev. Dis. Primers 6, 74. 10.1038/s41572-020-0205-x 32913180

[B23] Liang X. Wang X. He Y. Wu Y. Zhong L. Liu W. (2020). Acetylation Dependent Functions of Rab22a-NeoF1 Fusion Protein in Osteosarcoma. Theranostics 10, 7747–7757. 10.7150/thno.46082 32685017 PMC7359080

[B24] Liu L. Xu X. Zhang N. Zhang Y. Zhao K. (2020). Acetylase Inhibitor SI-2 Is a Potent Anti-inflammatory Agent by Inhibiting NLRP3 Inflammasome Activation. Int. Immunopharmacol. 87, 106829. 10.1016/j.intimp.2020.106829 32736194

[B25] Mak'anyengo R. Duewell P. Reichl C. Hörth C. Lehr H. A. Fischer S. (2018). Nlrp3-dependent IL-1β Inhibits CD103+ Dendritic Cell Differentiation in the Gut. JCI Insight 3, e96322. 10.1172/jci.insight.96322 PMC5922280 29515025

[B26] Mao L. Kitani A. Strober W. Fuss I. J. (2018). The Role of NLRP3 and IL-1β in the Pathogenesis of Inflammatory Bowel Disease. Front. Immunol. 9, 2566. 10.3389/fimmu.2018.02566 30455704 PMC6230716

[B27] Mariathasan S. Weiss D. S. Newton K. Mcbride J. O'rourke K. Roose-Girma M. (2006). Cryopyrin Activates the Inflammasome in Response to Toxins and ATP. Nature 440, 228–232. 10.1038/nature04515 16407890

[B28] Martinon F. Mayor A. Tschopp J. (2009). The Inflammasomes: Guardians of the Body. Annu. Rev. Immunol. 27, 229–265. 10.1146/annurev.immunol.021908.132715 19302040

[B29] Mei Y. Fang C. Ding S. Liu X. Hu J. Xu J. (2019). PAP-1 Ameliorates DSS-Induced Colitis with Involvement of NLRP3 Inflammasome Pathway. Int. Immunopharmacol. 75, 105776. 10.1016/j.intimp.2019.105776 31351364

[B30] Oike T. Komachi M. Ogiwara H. Amornwichet N. Saitoh Y. Torikai K. (2014). C646, a Selective Small Molecule Inhibitor of Histone Acetyltransferase P300, Radiosensitizes Lung Cancer Cells by Enhancing Mitotic Catastrophe. Radiother. Oncol. 111, 222–227. 10.1016/j.radonc.2014.03.015 24746574

[B31] Ono H. Basson M. D. Ito H. (2016). P300 Inhibition Enhances Gemcitabine-Induced Apoptosis of Pancreatic Cancer. Oncotarget 7, 51301–51310. 10.18632/oncotarget.10117 27322077 PMC5239476

[B32] Roda G. Chien Ng S. Kotze P. G. Argollo M. Panaccione R. Spinelli A. (2020). Crohn's Disease. Nat. Rev. Dis. Primers 6, 22. 10.1038/s41572-020-0156-2 32242028

[B33] Schmid-Burgk J. L. Chauhan D. Schmidt T. Ebert T. S. Reinhardt J. Endl E. (2016). A Genome-wide CRISPR (Clustered Regularly Interspaced Short Palindromic Repeats) Screen Identifies NEK7 as an Essential Component of NLRP3 Inflammasome Activation. J. Biol. Chem. 291, 103–109. 10.1074/jbc.C115.700492 26553871 PMC4697147

[B34] Swanson K. V. Deng M. Ting J. P.-Y. (2019). The NLRP3 Inflammasome: Molecular Activation and Regulation to Therapeutics. Nat. Rev. Immunol. 19, 477–489. 10.1038/s41577-019-0165-0 31036962 PMC7807242

[B35] Tourkochristou E. Aggeletopoulou I. Konstantakis C. Triantos C. (2019). Role of NLRP3 Inflammasome in Inflammatory Bowel Diseases. World J. Gastroenterol. 25, 4796–4804. 10.3748/wjg.v25.i33.4796 31543674 PMC6737309

[B36] Waljee A. K. Liu B. Sauder K. Zhu J. Govani S. M. Stidham R. W. (2018). Predicting Corticosteroid-free Endoscopic Remission with Vedolizumab in Ulcerative Colitis. Aliment. Pharmacol. Ther. 47, 763–772. 10.1111/apt.14510 29359519 PMC5814341

[B37] Wang Y. Tang Q. Duan P. Yang L. (2018). Curcumin as a Therapeutic Agent for Blocking NF-κB Activation in Ulcerative Colitis. Immunopharmacol. Immunotoxicol. 40, 476–482. 10.1080/08923973.2018.1469145 30111198

[B38] Wu Y. Chen H. Lu J. Zhang M. Zhang R. Duan T. (2015). Acetylation-dependent Function of Human Single-Stranded DNA Binding Protein 1. Nucleic Acids Res. 43, 7878–7887. 10.1093/nar/gkv707 26170237 PMC4652753

[B39] Zhao D. Fukuyama S. Sakai-Tagawa Y. Takashita E. Shoemaker J. E. Kawaoka Y. (2016). C646, a Novel p300/CREB-Binding Protein-specific Inhibitor of Histone Acetyltransferase, Attenuates Influenza A Virus Infection. Antimicrob. Agents Chemother. 60, 1902–1906. 10.1128/AAC.02055-15 PMC4776003 26711748

[B40] Zhao K. Zhang Y. Xu X. Liu L. Huang L. Luo R. (2019a). Acetylation Is Required for NLRP3 Self-Aggregation and Full Activation of the Inflammasome *.* 10.1101/2019.12.31.891556

[B41] Zhao Y. Guo Q. Zhu Q. Tan R. Bai D. Bu X. (2019b). Flavonoid VI-16 Protects against DSS-Induced Colitis by Inhibiting Txnip-dependent NLRP3 Inflammasome Activation in Macrophages via Reducing Oxidative Stress. Mucosal Immunol. 12, 1150–1163. 10.1038/s41385-019-0177-x 31152156

[B42] Zhen Y. Zhang H. (2019). NLRP3 Inflammasome and Inflammatory Bowel Disease. Front. Immunol. 10, 276. 10.3389/fimmu.2019.00276 30873162 PMC6403142

